# Treatment with Sotrovimab and Casirivimab/Imdevimab Enhances Serum SARS-CoV-2 S Antibody Levels in Patients Infected with the SARS-CoV-2 Delta, Omicron BA.1, and BA.5 Variants

**DOI:** 10.3390/idr14060099

**Published:** 2022-12-09

**Authors:** Kana Fujimoto, Satoru Mutsuo, Yuto Yasuda, Soichi Arasawa, Noriyuki Tashima, Daisuke Iwashima, Ken-ichi Takahashi

**Affiliations:** 1 Department of Respiratory Medicine, Kishiwada City Hospital, 1001 Gakuhara-cho, Kishiwada-shi 596-8501, Osaka, Japan; 2 Department of Central Clinical Laboratory, Kishiwada City Hospital, 1001 Gakuhara-cho, Kishiwada-shi 59-8501, Osaka, Japan; 3 Department of Gastroenterology, Kishiwada City Hospital, 1001 Gakuhara-cho, Kishiwada-shi 596-8501, Osaka, Japan

**Keywords:** COVID-19, SARS-CoV-2, S antibody, sotrovimab, remdesivir

## Abstract

Background: The neutralizing ability of sotrovimab and casirivimab/imdevimab against the severe acute respiratory syndrome coronavirus 2 (SARS-CoV-2) is attenuated in the subvariant BA.5. However, the efficacy of sotrovimab in the clinical setting remains to be investigated. Methods: Patients admitted to Kishiwada City Hospital with COVID-19 delta, omicron BA.1, or BA.5 subvariants were evaluated retrospectively for serum SARS-CoV-2 S and N antibody levels using the Elecsys Anti-SARS-CoV-2 assay. Results: In patients with COVID-19 during the BA.5 wave of the COVID-19 pandemic, anti-SARS-CoV-2 S antibody titers (median [interquartile range]) increased from 2154.0 (864.0–6669.3) U/mL on day 0 to 21,371.0 (19,656.3–32,225.0) U/mL on day 3 in the group treated with sotrovimab (*N* = 40) and were significantly higher than in the group treated with remdesivir plus dexamethasone plus baricitinib (*p* < 0.001). Conclusion: Treatment with sotrovimab could prevent severe disease in high-risk patients infected with SARS-CoV-2 subvariant BA.5.

## 1. Introduction

Sotrovimab is a monoclonal antibody that is available for the treatment of coronavirus disease 2019 (COVID-19) after receiving emergency use authorization in September 2021. Treatment with sotrovimab neutralizes sarbecoviruses, including severe acute respiratory syndrome coronavirus 2 (SARS-CoV-2), and reduces the risk of severe disease progression among high-risk patients with mild-to-moderate COVID-19 [1]. In Japan, approved treatments for mild COVID-19 include antibody therapies, such as sotrovimab and casirivimab/imdevimab [2], as well as antiviral drugs, such as remdesivir [3], molnupiravir [4], and nirmatrelvir/ritonavir [5]. Intravenously administered antibody-based therapies are especially useful in elderly patients who are more prone to developing severe disease [6] and often have swallowing dysfunction.

The SARS-CoV-2 omicron subvariant BA.5 has been the prevalent SARS-CoV-2 variant in Japan since July 2022, and is estimated to underlie >90% of all newly detected COVID-19 cases starting the fourth week of July. An in vitro study reported the attenuated effect of sotrovimab on the BA.5 subvariant [7]. FDA released a statement on 5 April 2022 that sotrovimab is no longer authorized to treat COVID-19 in any U.S. region, and WHO updated its guidelines on 16 September 2022 to strongly recommend against the use of sotrovimab in patients with non-severe COVID-19 [8,9]. However, sotrovimab had been used clinically due to its relative ease of use during the BA.5 wave of the COVID-19 pandemic at some hospitals in Japan.

In this study, we retrospectively evaluated the prognosis and analyzed SARS-CoV-2 S and N antibody levels in patients with COVID-19 during the BA.5 wave of the COVID-19 pandemic and compared them to the antibody levels in the BA.1 and delta waves of the pandemic.

## 2. Materials and Methods

### 2.1. Patients

We retrospectively analyzed patients with COVID-19 admitted to Kishiwada City Hospital (Osaka, Japan) during the waves of the COVID-19 pandemic, caused by the SARS-CoV-2 variants delta, omicron subvariant BA.1, and omicron subvariant BA.5. We estimated the variants based on the prevalent wave without variant sequencing. Namely, patients admitted between 24 July 2021 and 3 December 2021 were analyzed for the delta variant, 2 January 2022 and 23 March 2022 for the omicron subvariant BA.1, and 1 July 2022 and 10 August 2022 for the omicron subvariant BA.5. Clinical data were collected by reviewing patients’ medical charts. All laboratory data and chest anteroposterior X-ray images were obtained on the day of hospitalization. Chest X-ray abnormalities were evaluated by whether the extent of lung lesion was more than 50% or not.

### 2.2. Treatment

Patients who did not require oxygen therapy were treated with sotrovimab and casirivimab/imdevimab within 24 h of hospitalization. Treatment with remdesivir and dexamethasone with or without baricitinib (double or triple therapy) was initiated when the patients required oxygen therapy. Triple therapy was promptly initiated in patients that required oxygenation after sotrovimab treatment. The detailed protocol used for remdesivir therapy has been described previously [10].

### 2.3. Elecsys Anti-SARS-CoV-2 S and N Assay

Residual frozen serum samples from day 0 of patient hospitalization and from day 3 of treatment administration were analyzed. The Elecsys Anti-SARS-CoV-2 S and N assay (Roche, Basel, Switzerland) were performed according to the manufacturer’s instructions. The upper and lower limits of the S antibody titer were determined to be 100,000 and 0.4 U/mL, as per the manufacturer’s data sheet. The upper and lower limits of the N antibody (cut off index, COI) were not determined because all data were detectable.

### 2.4. Statistical Analysis

Continuous variable data in the study are expressed as mean ± standard deviation (SD) or median (interquartile range). The *p*-values of anti-SARS-CoV-2 S titers and COI of N antibody were calculated using two-way ANOVA with Sidak’s multiple comparison test and the Wilcoxon matched-pairs signed-rank test with a false discovery rate step-down procedure. The *p*-values of patient characteristics were calculated using the Student’s *t*-test, Mann–Whitney U test, or Fisher’s exact test. All statistical analyses of patient characteristics were performed using R version 4.2.1 and all statistical analyses of the experimental data were performed and visualized using GraphPad Prism, version 9.2.0 (GraphPad Software, San Diego, CA, USA). Statistical significance was set at *p* < 0.05.

## 3. Results

During the study period, 439 patients were admitted to the hospital with COVID-19, and residual sera were obtained from 179 patients on admission (day 0) and on the third day of treatment. All patients received treatment within 24 h of admission. The 179 patients analyzed in this study included 56 patients infected with the delta variant of SARS-CoV-2 (24 July–3 December 2021), 47 patients infected with the omicron subvariant BA.1 (2 January–23 March 2022), and 76 patients infected with the omicron subvariant BA.5 (1 July–10 August 2022).

### 3.1. Patient Characteristics during the Delta Wave of the COVID-19 Pandemic

Of the 56 patients infected with the delta variant of SARS-CoV-2, 25 patients received casirivimab/imdevimab therapy, 30 patients received remdesivir plus dexamethasone plus baricitinib (triple therapy), and one received remdesivir plus dexamethasone (double therapy). Two of the 25 patients treated with casirivimab/imdevimab therapy required oxygen administration on the first day of treatment, followed by triple therapy. Owing to the small number of cases of double and triple therapy after casirivimab/imdevimab, patients treated with casirivimab/imdevimab and triple therapy were included in the analysis.

As vaccination status affects antibody titers [11], patient characteristics according to vaccination status are summarized in Table 1. A total of 13 vaccinated and 10 unvaccinated patients received casirivimab/imdevimab therapy, and 4 vaccinated and 26 unvaccinated patients received triple therapy. Only one vaccinated patient treated with casirivimab/imdevimab was immunocompromised because of prior treatment with cyclosporin A. Two patients who received casirivimab/imdevimab therapy required oxygen upon admission, but did not require oxygen immediately before receiving casirivimab/imdevimab therapy. The duration from disease onset to hospitalization was significantly shorter in unvaccinated patients treated with casirivimab/imdevimab than in those treated with triple therapy. A similar trend was observed in vaccinated patients, but the difference was not statistically significant. All the patients recovered from COVID-19.

### 3.2. Patient Characteristics during the Omicron BA.1 Wave of the COVID-19 Pandemic

Of the 47 patients infected with the BA.1 omicron subvariant, 32 received sotrovimab therapy, 12 received triple therapy, and 3 received double therapy. Three of the 32 patients treated with sotrovimab required oxygen therapy on the first day of treatment, followed by triple or double therapy. Owing to the small number of cases of double and triple therapy after sotrovimab, patients treated with sotrovimab and triple therapy were included in the analysis. Fourteen patients with an unknown vaccination status were excluded from the study.

A total of 14 vaccinated and 2 unvaccinated patients received sotrovimab, and 10 vaccinated and 1 unvaccinated patient received triple therapy. Only one unvaccinated patient treated with sotrovimab was immunocompromised due to the use of several immune modulator drugs. In the vaccinated group, a higher flow of oxygen therapy was required in patients treated with triple therapy than in those treated with sotrovimab. Owing to the small number of cases, the unvaccinated group could not be analyzed. Patient characteristics are summarized in Table 2.

### 3.3. Patient Characteristics during the Omicron BA.5 Wave of the COVID-19 Pandemic

Of the 76 patients infected with the BA.5 omicron subvariant, 47 received sotrovimab therapy, 17 received triple therapy, and 12 received double therapy. Two of the 47 patients treated with sotrovimab required oxygen therapy on the first and third days of treatment, followed by double and triple therapy, respectively. Patients treated with sotrovimab, triple therapy, and double therapy were included in the analysis because of the small number of cases requiring additional therapy after sotrovimab. Five patients with an unknown vaccination status were excluded from the study.

A total of 40 vaccinated and 2 unvaccinated patients received sotrovimab therapy, 15 vaccinated and 1 unvaccinated patient received triple therapy, and 11 vaccinated patients received double therapy. Owing to the small number of cases, the unvaccinated group could not be analyzed. Patient characteristics are summarized in Table 3. Among the patients treated with sotrovimab, three were on prednisolone, and three had hematologic malignancies. In patients treated with triple therapy, one patient was on prednisolone and one was on methotrexate. Three patients who received sotrovimab therapy required oxygen treatment on admission but did not require oxygen immediately before receiving sotrovimab therapy.

### 3.4. Anti-SARS-CoV-2 Antibody Levelss and Prognosis

**Figure 1:**
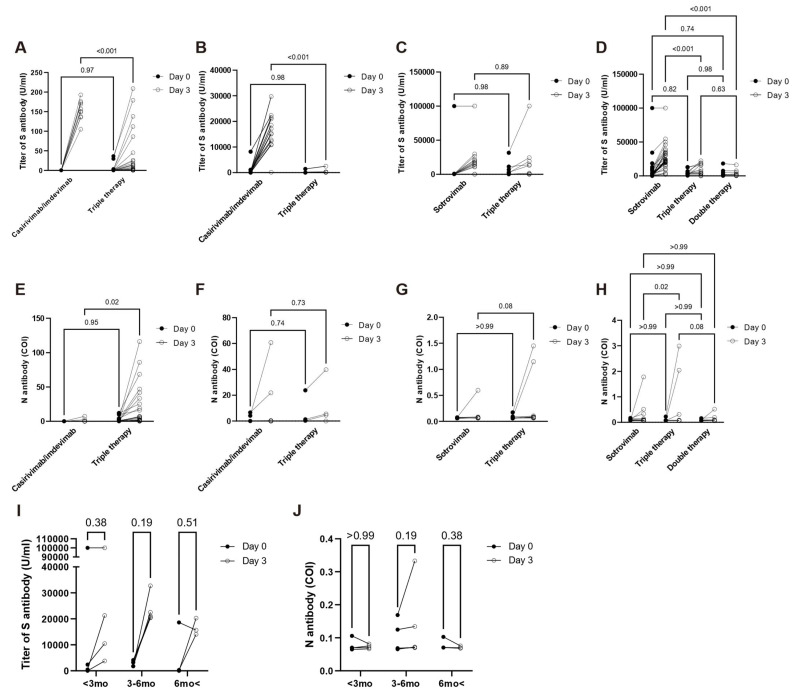
Titers of anti-SARS-CoV-2 S antibody were compared by therapy according to vaccination status and COVID-19 subvariant prevalence. ((**A**): unvaccinated-delta, (**B**): vaccinated-delta, (**C**): vaccinated-BA.1, and (**D**): vaccinated-BA.5). Levels of anti-SARS-CoV-2 N antibody (cut off index, COI) were compared by therapy according to vaccination status and COVID-19 subvariant prevalence. ((**E**): unvaccinated-delta, (**F**): vaccinated-delta, (**G**): vaccinated-BA.1, and (**H**): vaccinated-BA.5). Titers of anti-SARS-CoV-2 S antibody were compared between day 0 and day 3 according to last vaccination (<3 mo, within 3 months; 3–6 mo, between 3 and 6 months; 6 mo<, over 6 months) during the BA.5 wave. (**I**) Levels of anti-SARS-CoV-2 N antibody were compared between day 0 and day 3 according to last vaccination (<3 mo, within 3 months; 3–6 mo, between 3 and 6 months; 6 mo<, over 6 months) during the BA.5 wave. (**J**) *p*-Values were calculated using two-way ANOVA with Sidak’s multiple comparison test (**A**–**H**) and the Wilcoxon rank sum test and collected for multiple comparisons using a false discovery rate step down procedure (**I**,**J**).

We detected significantly higher anti-SARS-CoV-2 S antibody titers (S-Ab titers) on day 3 in patients treated with casirivimab/imdevimab against the delta strains than in those treated with triple therapy, regardless of vaccination. Patients treated with sotrovimab showed no significant difference in S-Ab titers compared to those treated with triple therapy during the BA.1 wave, but significantly higher S-Ab titers during the BA.5 wave compared to those treated with double or triple therapy (Figure 1A–D). Among vaccinated patients, the median S-Ab titer in patients treated with casirivimab/imdevimab against the delta strain on day 3 was 15,295.0 U/mL, and median S-Ab titers in patients treated with sotrovimab against the BA.1 and BA.5 strains were 17,042.5 and 21,371.0 U/mL, respectively. In contrast, median S-Ab titers in patients treated with triple therapy against the delta, BA.1, and BA.5 strains were 264.0 U/mL, 7347.5 U/mL, 5083.0 U/mL, respectively. Among the patients received both antibody and remdesivir treatment, the vaccinated and unvaccinated patients with COVID-19 delta experienced rapid surge of S-Ab (from 42.7 to 11,451 U/mL and from 0.4 to 167 U/mL, respectively). In the three BA.1 vaccinated patients, S-Ab increased rapidly from 119 to 23,077 U/mL, 576 to 17,210 U/mL, and 51.3 to 18,602 U/mL. In the two cases with BA.5, S-Ab increased rapidly from 134 to 31,363 U/mL and from 0.4 to 24,940 U/mL.

Significantly higher anti-SARS-CoV-2 N antibody (N-Ab) were detected in unvaccinated patients treated with triple therapy at the delta wave and in vaccinated patients treated with triple therapy at the BA.5 wave compared to those treated with casirivimab/imdevimab or sotrovimab (Figure 1E–H). A summary of these data is presented in Table 4.

Although information about COVID-19 vaccine type and number of administered doses was unavailable, we obtained detailed data on vaccination timing for 12 patients infected during the BA.5 wave. Three patients infected with COVID-19 were vaccinated 6 months prior to infection, five were vaccinated between 3 and 6 months, and four were vaccinated within 3 months of infection. Sotrovimab therapy increased S-Ab titers in all but one patient vaccinated 6 months prior to infection (Figure 1I); N-Ab levels are illustrated in Figure 1J.

An 85-year-old patient who received triple therapy during the BA.1 wave, a 79-year-old patient treated with sotrovimab, and an 88-year-old patient treated with double therapy during the BA.5 wave died during COVID-19 infection. However, the cause of death was determined to be either terminal cancer or aspiration pneumonia. All other patients recovered from COVID-19 without requiring intensive care.

## 4. Discussion

In this study, we report significantly higher anti-SARS-CoV-2 S antibody (S-Ab) levels in patients treated with sotrovimab after infection with the SARS-CoV-2 subvariant BA.5. compared to double or triple therapy. To the best of our knowledge, this is the first report to demonstrate the efficacy of sotrovimab in increasing S-Ab levels in real clinical practice.

Sotrovimab and casirivimab/imdevimab were neutralizing antibodies targeting the spike protein of SARS-CoV-2. Previous studies have reported attenuated in vitro activity of sotrovimab [7]. Interestingly, the rapid surges of S-Ab titers were still observed in the patients treated with sotrovimab during the BA.5 wave (from 2154.0 to 21,371.0 U/mL), as with in the vaccinated patients treated with casirivimab/imdevimab during the delta wave (from 301.0 to 15,295.0 U/mL). A previous study reported delayed antibody production in lethal COVID-19 [12]. In addition, early expression of endogenous antibodies may contribute to a reduction in disease severity [13]. Thus, a rapid surge in S-Ab titers may be important to prevent severe disease and death in COVID-19 patients. Sotrovimab might be effective against SARS-CoV-2 subvariant BA.5.

Vaccination against COVID-19 is important to reduce mortality due to COVID-19 infection [14]. During the delta wave of the COVID-19 pandemic, vaccinated patients had median S-Ab titers of 15,295.0 U/mL, whereas unvaccinated patients had a titer of only 154.0 U/mL. The increased antibody titers may result in reduced mortality, as previous studies have demonstrated a correlation between the Elecsys anti-SARS-CoV-2 S assay titer and neutralizing antibody response [15]. However, we could not investigate the correlation between patient mortality and S-Ab titers in our study due to few deaths in our study population. Infection with the omicron variant causes less severe disease than the delta variant [16], and the risk of severe disease and death is similar in the BA.4/BA.5 and BA.1 waves [17], making it difficult to compare mortality.

Information about the type and date of COVID-19 vaccination was not available for most of the patients included in our study. An observational study, stratified by time since vaccination found that the efficacy decreased from 42% to 57% for mRNA vaccines and 47.3% for AZD1222 4–6 months after vaccination [18]. Based on the mass vaccination schedule in Japan, most patients infected with BA.5 were considered to be immunized for more than 3 months since their last vaccination. Although the small number of cases made it difficult to correlate S-Ab titers with the time since the last vaccination, sotrovimab may be effective even in patients whose last vaccination was over 6 months prior to infection.

Most patients in our study had a low level of Anti-SARS-CoV-2 N antibody (N-Ab). N-Abs are an important indicator of natural infections. N-Ab positivity was reported in 51.2% vaccinated individuals and 98.8% infected patients [19]. Therefore, most of the subjects in this study were not considered to have been infected in the past. The neutralizing effects of N-Abs remain unknown. Patients who required oxygen therapy during the delta wave had higher N-Ab values, but the clinical significance of increased N-Ab values was unclear. There are not enough reports regarding levels of N-Ab. The reason for this is still unclear and requires further investigation.

This study had several limitations that need to be considered. First, this study was retrospective and only a small set of selected data was analyzed. A larger data set would help to identify the factors that influence S-Ab and N-Ab levels. Second, the SARS-CoV-2 variant was estimated based on the prevalent wave, and variant sequencing was not performed. Third, we did not investigate other treatments for mild COVID-19. Moreover, in vaccinated patients, a rapid S-Ab surge may be observed without any treatment. We chose the triple therapy group as the control, and baricitinib or dexamethasone might reduce the S-Ab titer at day 3. High-dose steroids have been reported to decrease immunoglobulins by an average of 22%, although no significant difference in the S antibody titer was observed between corticosteroid-treated (250–500 mg/day) and untreated patients with COVID-19 [20]. Further randomized larger studies are required to confirm the efficacy of sotrovimab for other omicron subvariants.

## 5. Conclusions

Sotrovimab may be an effective therapeutic for the prevention of severe disease in high-risk patients infected with the COVID-19 subvariant BA.5.

## Figures and Tables

**Table 1:** Patient characteristics during the delta wave.

	Vaccinated			Unvaccinated		
Characteristic	Casirivimab/Imdevimab, *N* = 13	Triple Therapy, *N* = 4	*p*-Value	Casirivimab/Imdevimab, *N* = 10	Triple Therapy, *N* = 26	*p*-Value
Age, mean ± SD	65.5 ± 20.5	53.2 ± 3.3	0.2	50.8 ± 13.5	51.8 ± 11.9	>0.9
Sex (Female/Male)	5/8	1/3	>0.9	2/8	12/14	0.3
Smoking History (Yes/No/Unknown)	6/7/0	3/1/0	0.6	6/4/0	14/12/0	>0.9
Previous history of diabetes, *N* (%)	4 (31%)	2 (50%)	0.6	0 (0%)	4 (15%)	0.6
Previous history of ischemic heart disease, *N* (%)	1 (7.7%)	0 (0%)	>0.9	1 (10%)	0 (0%)	0.3
Lymphocyte count, /µL	1260.9 ± 402.5	802.2 ± 279.5	0.045	917.7 ± 449.0	1013.2 ± 402.7	0.3
WBC, /µL	5490.0 ± 1703.0	6507.5 ± 695.8	0.2	4394.0 ± 1949.0	5253.8 ± 1672.3	0.3
CRP, mg/dL	3.3 ± 4.4	10.2 ± 4.8	0.036	2.0 ± 2.0	8.0 ± 4.6	<0.001
D-dimer, mg/dL	1.4 ± 0.9	2.1 ± 1.1	0.066	0.9 ± 0.4	1.4 ± 0.3	<0.001
Oxygen therapy at hospitalization, L/min	0.1 ± 0.3	3.0 ± 1.4	<0.001	0.2 ± 0.6	1.8 ± 1.2	<0.001
Chest X-ray abnormalities (>50%/<50%/0%)	0/10/3	2/2/0	0.05	0/5/5	6/19/1	0.004
Duration from onset to treatment	2.7 ± 1.2	6.5 ± 4.0	0.12	2.9 ± 2.0	7.0 ± 2.2	<0.001

SD, standard deviation; WBC, white blood cell; CRP, c-reacting protein.

**Table 2:** Patient characteristics during the BA.1 wave.

	Vaccinated		
Characteristic	Sotrovimab, *N* = 14	Triple Therapy, *N* = 10	*p*-Value
Age, mean ± SD	72.1 ± 21.6	77.5 ± 13.0	0.7
Sex (Female/Male)	6/8	3/7	0.7
Smoking History (Yes/No/Unknown)	7/5/2	6/4/0	0.7
Previous history of diabetes, *N* (%)	2 (14%)	2 (20%)	>0.9
Previous history of ischemic heart disease, *N* (%)	1 (7.1%)	2 (20%)	0.6
Lymphocyte count, /µL	1177.6 ± 391.8	1021.5 ± 488.2	0.3
WBC, /µL	5442.1 ± 2412.6	5732.0 ± 1704.7	0.3
CRP, mg/dL	2.6 ± 3.4	5.8 ± 5.7	0.079
D-dimer, mg/dL	1.1 ± 0.5	1.6 ± 1.0	0.13
Oxygen therapy at hospitalization, L/min	0.0 ± 0.0	2.0 ± 1.6	<0.001
Chest X-ray abnormalities (>50%/<50%/0%)	0/6/8	1/9/0	0.006
Duration from onset to treatment	2.1 ± 0.8	3.8 ± 3.3	0.4

SD, standard deviation; WBC, white blood cell; CRP, c-reacting protein.

**Table 3:** Patient characteristics during the BA.5 wave.

	Vaccinated			
Characteristic	Sotrovimab, *N* = 40	Triple Therapy, *N* = 15	Double Therapy, *N* = 11	*p*-Value
Age, Mean ± SD	79.2 ± 9.9	84.5 ± 9.7	82.5 ± 15.2	0.15
Sex (Female/Male)	22/18	11/4	7/4	0.5
Smoking History (Yes/No/Unknown)	15/25/0	5/10/0	6/5/0	0.5
Previous history of diabetes, *N* (%)	10 (25%)	3 (20%)	1 (9.1%)	0.6
Previous history of ischemic heart disease, *N* (%)	12 (30%)	5 (33%)	5 (45%)	0.7
Lymphocyte count, /µL	1008.5 ± 470.3	692.2 ± 332.9	852.8 ± 591.7	0.036
WBC, /µL	6465.5 ± 3549.2	5998.0 ± 1726.8	7188.2 ± 3230.6	0.7
CRP, mg/dL	4.3 ± 6.0	4.2 ± 3.9	4.4 ± 3.6	0.6
D-dimer, mg/dL	3.9 ± 15.8	2.2 ± 1.9	3.6 ± 3.0	0.017
Oxygen therapy at hospitalization, L/min	0.2 ± 0.6	2.3 ± 1.3	2.8 ± 1.5	<0.001
Chest X-ray abnormalities (>50%/<50%/0%)	2/13/25	3/10/2	3/8/0	<0.001
Duration from onset to treatment	1.5 ± 1.0	2.0 ± 2.2	1.6 ± 1.3	>0.9

SD, standard deviation; WBC, white blood cell; CRP, c-reacting protein.

**Table 4:** Titers of anti-SARS-CoV-2 S and N antibody according to treatment and vaccination status.

	S Antibody (U/mL)						N Antibody (Cut off Index)					
	Day 0			Day 3			Day 0			Day 3		
	Median	Q1	Q3	Median	Q1	Q3	Median	Q1	Q3	Median	Q1	Q3
**Unvaccinated-delta**												
Casirivimab/imdevimab (*N* = 13)	0.4	0.4	0.4	154.0	136.8	173.8	0.06	0.06	0.12	0.08	0.06	6.86
Triple therapy (*N* = 4)	0.4	0.4	1.4	7.8	0.7	31.0	0.23	0.06	10.44	5.24	0.35	73.78
**Vaccinated-delta**												
Casirivimab/imdevimab (*N* = 10)	301.0	10.7	689.0	15,295.0	11,649.0	21,268.5	0.06	0.06	5.91	0.07	0.06	49.06
Triple therapy (*N* = 26)	176.5	49.0	1129.8	264.0	113.7	1972.3	0.81	0.06	23.80	4.99	0.06	39.80
**Vaccinated-BA.1**												
Sotrovimab (*N* = 14)	223.5	122.0	409.5	17,042.5	11,529.3	22,518.0	0.06	0.06	0.08	0.07	0.06	0.34
Triple therapy (*N* = 10)	1220.0	141.5	7967.0	7347.5	384.0	20,528.0	0.06	0.06	0.16	0.09	0.06	1.42
**Vaccinated-BA.5**												
Sotrovimab (*N* = 40)	2154.0	864.0	6669.3	21,371.0	19,656.3	32,225.0	0.07	0.07	0.11	0.07	0.07	0.13
Triple therapy (*N* = 15)	3767.0	500.0	6754.0	5083.0	2278.0	16,847.0	0.07	0.06	0.14	0.07	0.07	2.43
Double therapy (*N* = 11)	1864.0	303.0	5001.0	2190.0	637.0	5437.0	0.07	0.06	0.15	0.07	0.07	0.45

## Data Availability

The datasets generated and/or analysed during the current study are available from the corresponding author on reasonable request.
